# Is Serum Ferritin a Predictor of Blood Transfusions Outcome and Survival in Childhood Lymphomas and Solid Tumors?

**DOI:** 10.3390/cancers16223742

**Published:** 2024-11-06

**Authors:** Małgorzata Sawicka-Żukowska, Anna Krętowska-Grunwald, Magdalena Topczewska, Maryna Krawczuk-Rybak, Kamil Grubczak

**Affiliations:** 1 Department of Pediatric Oncology and Hematology, Medical University of Bialystok, Jerzego Waszyngtona 17, 15-274 Bialystok, Poland; annammkretowska@gmail.com (A.K.-G.); rybak@umb.edu.pl (M.K.-R.); 2 Department of Regenerative Medicine and Immune Regulation, Medical University of Bialystok, Jerzego Waszyngtona 13, 15-269 Bialystok, Poland; kamil.grubczak@umb.edu.pl; 3 Faculty of Computer Science, Bialystok University of Technology, Wiejska 45A, 15-351 Bialystok, Poland; m.topczewska@pb.edu.pl

**Keywords:** ferritin, iron overload, childhood lymphoma, childhood solid tumors, blood transfusion

## Abstract

Packed red blood cell (PRBC) transfusions are an important part of supportive treatment in oncology; however, when used frequently, they can be a result of transfusion-related iron overload. The aim of the study was to evaluate the role of ferritin as a non-specific marker of neoplastic growth and transfusion-related iron overload in children with lymphomas and solid tumors. We performed a longitudinal analysis of PRBC transfusions and changes in ferritin concentrations during oncological treatment of 88 children with lymphomas and solid tumors. A ferritin concentration above 500 ng/mL was diagnosed in 14.77% of patients at the moment of admission and 18.18% at the end of treatment. No differences were shown in serum ferritin in the context of tumor type-, sex-, and transfusion-related parameters. Those above the age of 10 demonstrated higher ferritin concentrations compared to subjects younger than 5 years of age. In addition, those over than 10 years old or above 30 kg in weight showed a tendency for better survival. All tested patients demonstrated highly significant correlations between ferritin at the 15th month of treatment or after therapy discontinuation and transfusion-related parameters. Interestingly, ferritin levels were found to lower back to the values before therapy shortly after its discontinuation. Transfusion parameters and ferritin levels had no influence on the survival of the studied cancer patients.

## Simple Summary

Post-transfusion iron overload is a common side effect of anticancer treatment of childhood malignancies. We showed that serum ferritin levels could be valuable prognostic marker of iron overload in children with solid tumors and lymphomas. Interestingly, ferritin levels were found to lower back to the values before therapy shortly after its discontinuation. Transfusion parameters and ferritin levels had no influence on the survival of the studied cancer patients.

## 1. Introduction

Ferritin is a 450 kDa protein mainly derived from macrophages which is composed of two functionally and genetically different subunits—L-ferritin (light chain) and H-chain (heavy chain) [1,2,3,4]. Ferritin is abundant in circulation, being the oldest known protein involved in iron metabolism as a reliable indicator of iron storages [1,2,3]. In addition, ferritin plays a role as an acute-phase protein, and serum ferritin concentrations are elevated during the acute and chronic phases of the inflammatory process. Besides iron metabolism and inflammation, serum ferritin plays an important role in cancer, including immunosuppression, angiogenesis, and iron-independent cell proliferation [1,2,4,5,6,7]. Recent studies have brought novel insights into ferritin’s involvement in ferritinophagy and ferroptosis [5,6]. Ferritin has been found to be overexpressed in tissues of multiple malignancies, including breast cancer, pancreatic cancer, hepatocellular carcinoma, glioblastoma, prostate cancer, and Hodgkin lymphoma [4,8,9,10,11,12,13,14]. Abnormal increases in L-ferritin in the tissues of selected tumors have been connected with aggressiveness, enhanced cancer cell proliferation, and a protective role against chemotherapy, resulting in worse prognoses [1,2,5,15].

Serum ferritin (SF) concentration also has a potential role as a marker of disease activity in some childhood malignancies, including leukemias, Hodgkin and non-Hodgkin lymphomas, and solid tumors. An evaluation of multiple SF levels during cancer therapy showed that a return to normal concentration was associated with a good response to treatment [10,11,13,16,17,18,19,20,21,22].

The evaluation of serum ferritin levels in cancer patients requires taking into account multiple functions of ferritin and changes in serum ferritin under diverse conditions, including primary advancement of the disease, coexisting inflammatory processes, increasing iron burden due to multiple transfusions, or therapy response [1,15,23,24,25,26,27,28]. B-symptoms in Hodgkin lymphomas (HLs) reflect the inflammatory response as an important component of the disease [14,21,29]. On the other hand, SF levels in HL patients are considered to be a predictor of disease activity and relapse due to dysregulation in iron metabolism and overexpression of ferritin in cancer tissue. Blood product transfusions in HL mainly concern patients in higher stages due to intensive treatment, and are rare in lower stages [21,29].

Advanced solid tumors and lymphomas in children require multiple blood product transfusions due to diverse causes, including bone marrow infiltration by the disease and metastases, myelosuppression due to chemotherapy and radiotherapy, diminished production of blood cells caused by chronic disease, blood loss, and malnutrition leading to nutritional deficiencies. Some of these patients require stem cell auto- or allotransplants preceded by high-dose chemotherapy, which can lead to increased demand for transfusions.

Blood product transfusions are inseparably connected with the anticancer therapy as an important part of supportive treatment. Although PRBC transfusion requirements in childhood solid tumors are limited to specific clinical and laboratory parameters, the intensity of transfusions in advanced cancer stages can lead to iron overload. Compared to children treated for leukemias, the number of transfusions in patients treated for solid tumors is incomparably lower, although serum ferritin concentration is considered as an objective marker of post-transfusion iron burden in both leukemic and non-leukemic patients [27,30,31,32,33,34,35].

To date, there are no comprehensive data on serum ferritin’s contribution to the blood transfusion-related changes in the course of cancer management. Furthermore, there is a knowledge gap regarding ferritin’s potential influence on the outcome of therapy and patients’ survival. The aim of this study was to evaluate the prognostic role of serum ferritin concentration in children with lymphomas and solid tumors. We also determined the changes in SF levels during antineoplastic treatment due to post-transfusion iron burden. Additional attention was paid to evaluating the clinical utility of transfusion-related parameters to estimate the risk of iron overload in pediatric solid tumors and lymphomas.

## 2. Materials and Methods

### 2.1. Patients

The study population was selected from patients with lymphomas and solid tumors during the period between subsequent stages of therapy from the Department of Pediatrics, Oncology, and Hematology of Medical University of Bialystok, Poland. They had received more than one blood transfusion. The study was conducted retrospectively. Inclusion criteria were: subjects not during active chemotherapy at the moment of evaluation, aged between 0 and 18 years, diagnosis of malignant solid tumor or lymphoma, and no medical history of diagnosed genetic iron metabolism disorder before or during oncological therapy. Exclusion criteria were: patients who underwent only one blood transfusion throughout treatment and all patients with oncological diseases other than lymphoma, age above 18 years, second malignancy, history of using chelator treatment for iron overload, and those with no laboratory and clinical signs of infection. The data were evaluated from patients’ transfusions books, physical examinations, and medical documentation, starting from January 2010 until December 2020. The following variables were collected from all patients: demographics (age and gender) and clinical diagnosis (diagnosis, date of diagnosis). After excluding patients who did not meet the inclusion criteria, the remaining 88 participants (male: 53, female: 35) between 1 month and 18 years of age (median age 3.9 years) were analyzed. We performed precise analysis in subgroups according to diagnosis—lymphomas (*n* = 22) and solid tumors (*n* = 66). The patients’ characteristics are exhibited in Table 1, including the age distribution in total and in both the lymphoma and solid tumor groups, as well as detailed descriptions of the diagnoses. The study was approved by Local Bioethical Committee, with the permission number: APK.002.36.2021. Informed consent was obtained from all the study subjects or legal guardians.

### 2.2. Laboratory Tests

Serum ferritin (SF, ng/mL), used as a marker of iron overload (IO), was evaluated using the electrochemiluminescence method (our hospital reference values: 15–150 ng/mL) in every patient at different stages of therapy: at the beginning of the treatment (single measurement—SF at baseline), during the course of the treatment (5 separated measurements during the treatment depending on the time of the therapy TP1-TP5 (time point, TP); mean interspace between each subsequent point was 2–4 months), and after the cessation of therapy (single measurement at the end of treatment—SF at finish). The cutoff categories describing IO according to elevated (>500 ng/mL) and significantly elevated (>1000 ng/mL) SF are widely used in the literature to assess the risk of post-transfusion IO [24,36,37]. All of the measurements were performed in the local laboratory at the Children University Hospital in Bialystok.

All the baseline characteristics were collected from medical records, including age, sex, diagnosis, and serum ferritin concentration (ng/mL). The diagnosis of neoplastic disease was based upon an evaluation of laboratory tests such as blood tests, tumor biopsies, radiological imaging, bone marrow and bone biopsies, and immunophenotype. The laboratory data were collected within the first 24 h after the admission.

### 2.3. Blood Transfusion

The level of hemoglobin at which PRBCs (packed red blood cells) were administered was below 8 g/dL, taking into account the present clinical symptoms of anemia and the age of the child. In most patients, transfusion was performed in cases of lower than 8 g/dL. The parameters qualifying patients for blood transfusion were measured in each patient as follows: (A) total amount of received blood in milliliters (during whole treatment process), (B) total amount of received blood per kilogram of body weight, and (C) total number of received transfusion units, i.e., episodes of transfusion.

### 2.4. Statistical Analysis

The acquired data were analyzed with the use of GraphPad Prism 9.0 software (GraphPad Prism Inc., Boston, MA, USA). The normality of the data distribution was verified using the Shapiro–Wilk, Anderson–Darling, and D’Agostiono and Pearson tests before the application of specific tests. Differences between unpaired groups were analyzed using the nonparametric Mann–Whitney test. The significance of the changes reported over the course of therapy monitoring was established with two-way ANOVA and Fisher’s LSD test. Ferritin was correlated with clinical parameters with the application of the nonparametric Spearman correlation test. Chi-square tests were used to evaluate differences in the distribution of selected parameters among the tested groups. The log-rank (Mantel–Cox) test was applied in survival analysis, and the median overall survival is presented. The significance level was set at *p* < 0.05, which is indicated on the graphs as the exact *p* value or asterisks: *—*p* < 0.05, **—*p* < 0.01, ***—*p* < 0.001, ****—*p* < 0.0001.

## 3. Results

### 3.1. Ferritin Levels Before Therapy in Lymphomas and Solid Tumors

**Figure 1:**
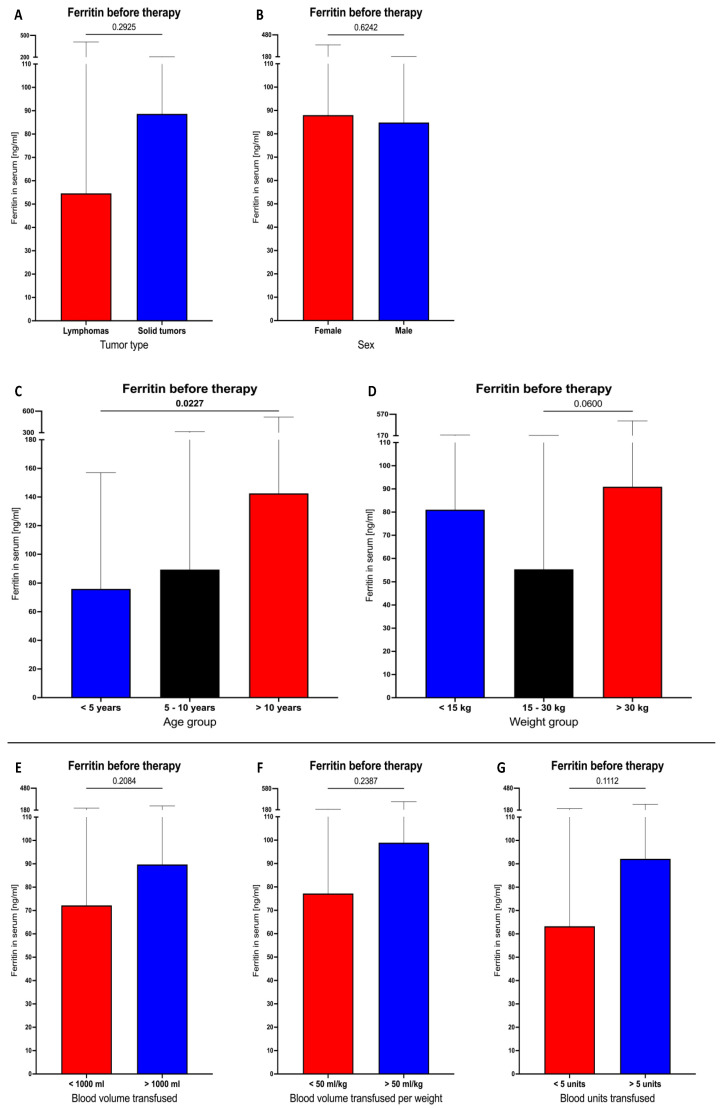
Pre-treatment blood ferritin levels in studied lymphoma and solid tumor patients. Initial ferritin concentrations between the total group of lymphoma and solid tumor subjects (**A**), additionally including stratification based on sex (**B**), age (**C**), or weight (**D**). Transfusion-related parameters’ influence on ferritin concentration: total volume of blood used (**E**), volume of transfused blood per kilogram (**F**), units of blood transferred (**G**). Data are presented as median values and 25th–75th percentile.

We did not observe any differences between lymphoma and solid tumor subjects in the context of blood ferritin levels before therapy implementation. The same results were obtained when all patients were stratified on the basis of gender. Significantly higher basal ferritin levels were demonstrated in patients older than 10 years old compared to those below 5 years old (*p* = 0.0227). A slight tendency for a higher ferritin level was shown in patients with weight above 30 kg compared to those weighing 15–30 kg (*p* = 0.0600) (Figure 1A–D). Next, we assessed whether transfusion settings had any effect on the ferritin levels observed before therapy. In accordance, no differences were shown in the studied patients considering the total volume of blood transfused, the volume per kg of subjects’ weight, or the total units of blood used for transfusion (Figure 1E–G).

### 3.2. Therapy Application Effect on Ferritin Concentration in Studied Patients

**Figure 2:**
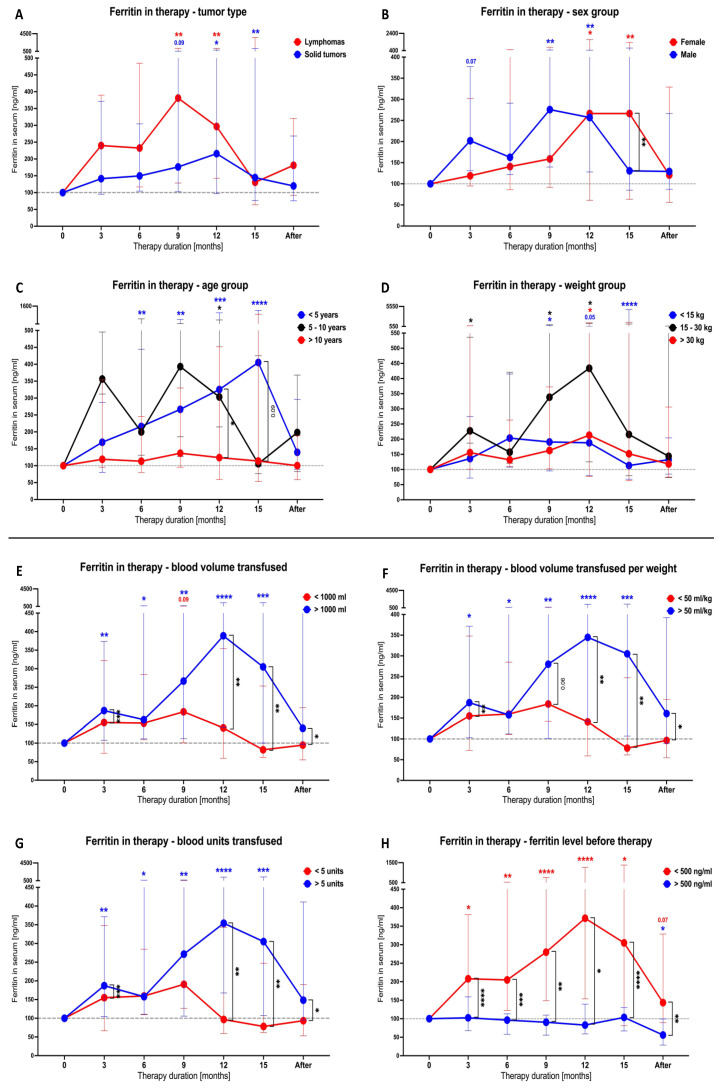
Therapy-induced variations in ferritin level in studied cancer patients. Changes in serum ferritin levels in total lymphoma and solid tumor groups (**A**), additionally including stratification based on sex (**B**), age (**C**), and weight (**D**). Transfusion-related parameters’ influence on ferritin concentration: total volume of blood used (**E**), volume of transfused blood per kilogram (**F**), units of blood transferred (**G**), and pre-treatment ferritin levels (**H**). Data are presented as median values and 25th–75th percentile, showing the percentage of ferritin change over time from the first time point (0 months). Statistical significance indicated with asterisks or *p* value. *—*p* < 0.05, **—*p* < 0.01, ***—*p* < 0.001, ****—*p* < 0.0001.

Next, we assessed the impact of the applied treatment on the SF concentration in the studied patient groups (Table 2). Implementation of therapy led to a gradual increase in blood ferritin levels, reaching the highest values at around the 9th or 12th month in lymphoma (*p* = 0.0026) and solid tumor (*p* = 0.0102) patients, respectively. Although solid tumor subjects still demonstrated higher ferritin values at the 15th month of treatment compared to those before therapy, the discontinuation of treatment diminished the changes observed at previous time points (Figure 2A). Male patients were found to reach the highest ferritin point at the 9th month of treatment (*p* = 0.0049) and maintained it at this level until the 12th (*p* = 0.0025), whereas females showed a comparable elevation between the 12th and 15th month (*p* = 0.0165 and *p* = 0.0016, respectively). Due to the earlier decline in the SF level in male subjects, females demonstrated significantly higher values of ferritin (*p* = 0.0026) at the 15th month of therapy (Figure 2B). Age-based stratification showed that subjects younger than 5 years old had the most stable increase in ferritin concentration in the course of therapy, with the first significant levels observed at the 6-month time point (*p* = 0.0093) and the highest value at the 15th month (*p* < 0.0001). Interestingly, no substantial changes were demonstrated in patients older than 10 years old, and the concentrations were significantly lower than those in the younger patient groups around the 12th to 15th month of treatment (*p* = 0.0324) (Figure 2C). The weight of the subjects did not seem to exert a crucial effect on the ferritin variations in the course of therapy, with all subgroups reaching the highest levels between the 9th and 12th months of treatment. Those with a weight of 15 to 30 kg showed the highest increase in ferritin, especially at the 12-month time point (*p* = 0.0137) (Figure 2D).

Furthermore, we verified whether transfusion-related parameters had an influence on the response to therapy reflected by changes in blood ferritin levels. First, we found that only subjects with total blood volume transfused above 1000 mL showed gradual increases in ferritin, starting with significant values at the 3rd (*p* = 0.0039) month and reaching the maximum at the 12th month of therapy (*p* < 0.0001). Subsequently, the ferritin concentration declined, leading to values comparable to pre-treatment detected at the last measured time point, after therapy discontinuation (Figure 2E). Comparable results were achieved in the context of blood volume per kilogram or total blood units transfused. Importantly, clear differences between those with higher volumes/units of blood transfused versus the opposite subgroup appeared around the 12th (*p* = 0.0010) to 15th month of therapy (*p* = 0.0020) (Figure 2F,G). Stratification of the patients into those with initial ferritin concentrations below and above 500 ng/mL showed crucial variations in the course of therapy. Subjects with pre-treatment ferritin lower than 500 ng/mL showed substantially higher rates of response, with constant growth in the tested parameter until the 12–15th month. No changes in ferritin were demonstrated in patients with basal levels higher than 500 ng/mL, causing a significant difference versus the opposite group at every point of therapy monitoring (Figure 2H).

### 3.3. Association of Ferritin Levels with Transfusion-Related Clinical Data of Lymphoma and Solid Tumor Patients

**Figure 3:**
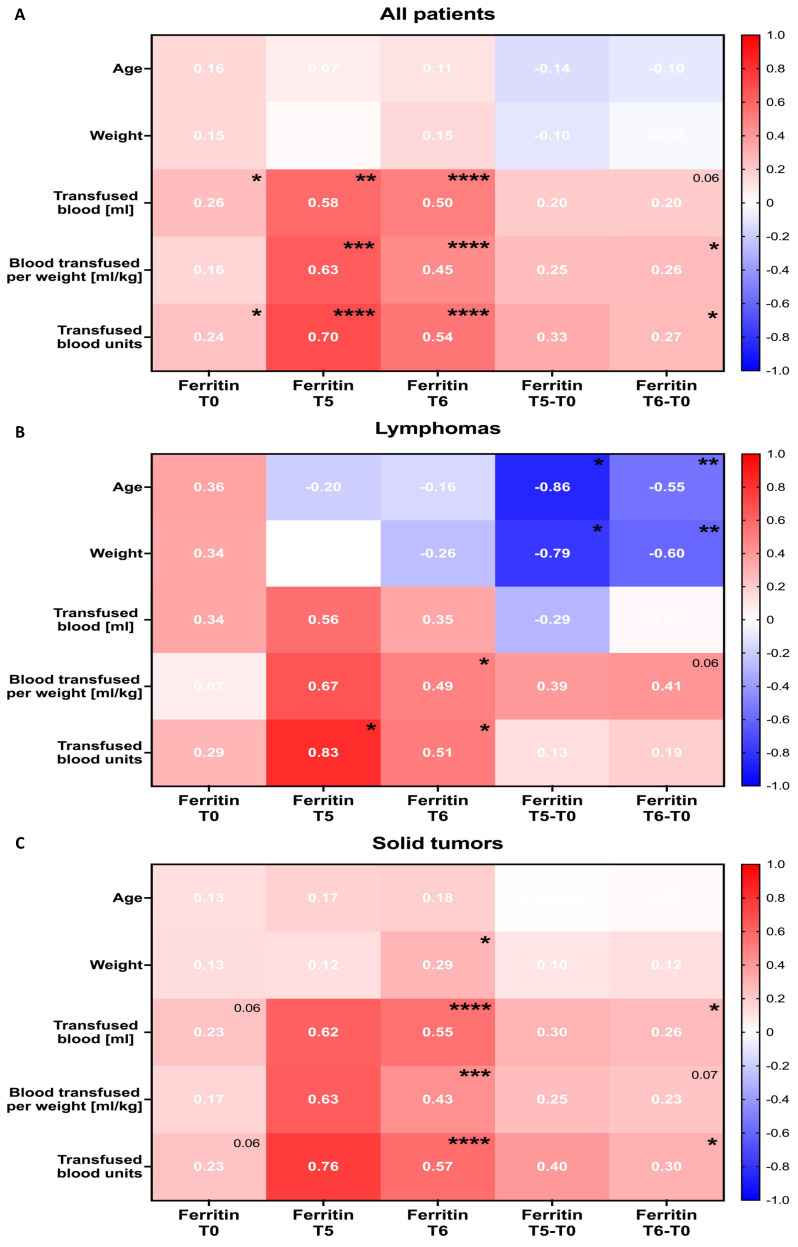
Correlations between ferritin levels in therapy and transfusion-related data in studied patients. Evaluated time points included: 0 (T0) and 15 (T5) months, the after-therapy point (T6), and the change between those periods. Correlations were assessed within the total group of patients (**A**) and in lymphoma (**B**) and solid tumor (**C**) patients individually. Data are presented on heat-maps as correlation coefficient (*r*) values, and statistical significance is indicated with asterisks or *p* value. *—*p* < 0.05, **—*p* < 0.01, ***—*p* < 0.001, ****—*p* < 0.0001.

All tested patients demonstrated highly significant correlations between ferritin at the 15th (T5) month of treatment or after therapy discontinuation (T6), and the transfusion-related parameters included total blood volume transfused (*p* < 0.05), volume used per kilogram (*p* < 0.05), and total blood units transfused (*p* < 0.05). Noteworthily, stronger associations were reported in reference to the T0 ferritin concentrations versus those obtained from T6 time point analysis (moderate correlations). Other comparisons, even significant ones, showed only weak associations between the tested ferritin levels and the transfusion parameters. No substantial results were obtained in context of weight or age correlation with ferritin values (Figure 3A).

Individual analysis of lymphoma subjects, despite not being statistically significant for all comparisons, showed strong or moderate associations between ferritin at the T5 and T6 time points and transfusion-related data. Importantly, we found that changes in ferritin levels demonstrated moderate to strong negative correlations with the age and weight of the lymphoma patients. This was especially evident when analyzing the change in ferritin between the 15th month and pre-treatment time points (Figure 3B). In reference to solid tumor patients, we showed the correlation results to be comparable to those reported when the total patients were analyzed (Figure 3C).

### 3.4. Distribution of Relapse and Death Incidents in Studied Lymphoma and Solid Tumor Patients

**Figure 4:**
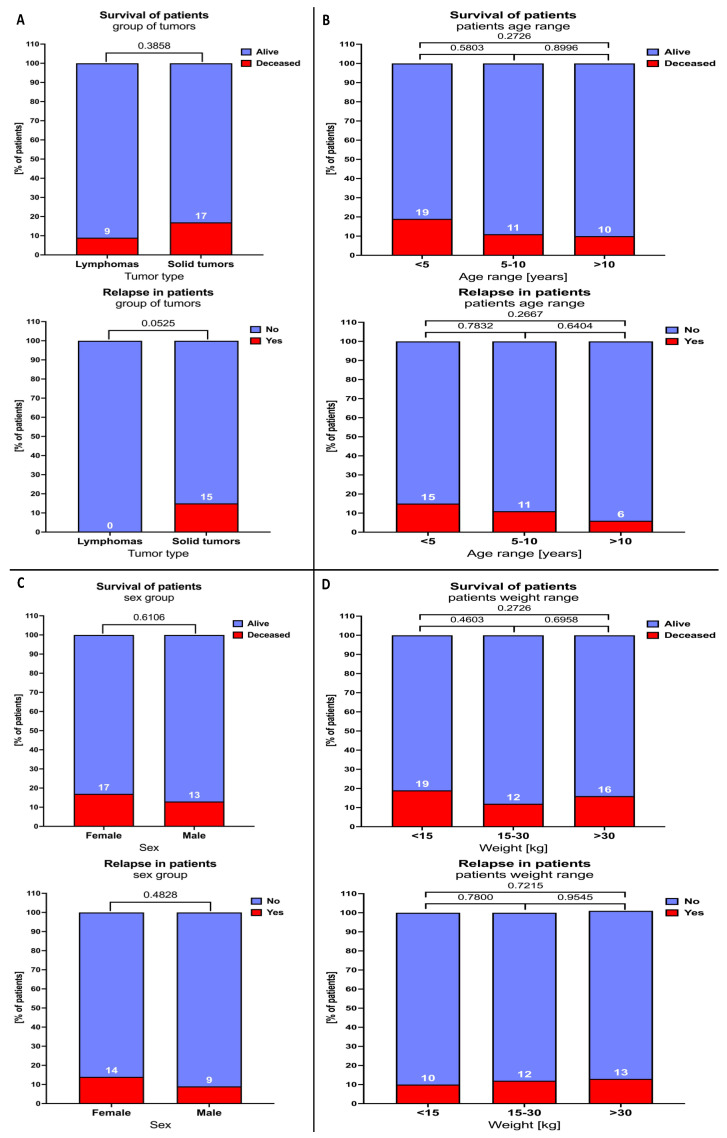
Distribution of relapse and death incidents in lymphoma and solid tumor patients. Chi-square analysis was performed individually, including tumor type (**A**), age (**B**), sex (**C**), and weight (**D**). Data show the frequency of death/relapse incidence in specific subgroups. Data significance is indicated with exact *p* values.

Firstly, we did not find any significant differences in the deceased/relapsed subjects‘ distribution between studied patients, including age-, sex-, and weight-based stratification. A close tendency for significance was demonstrated in reference to relapse incidence between different tumor type groups. In the solid tumor patients group, we reported the highest frequency of relapsed patients (15%) compared to the lymphoma subjects (0%) (*p* = 0.0525) (Figure 4A–D).

**Figure 5:**
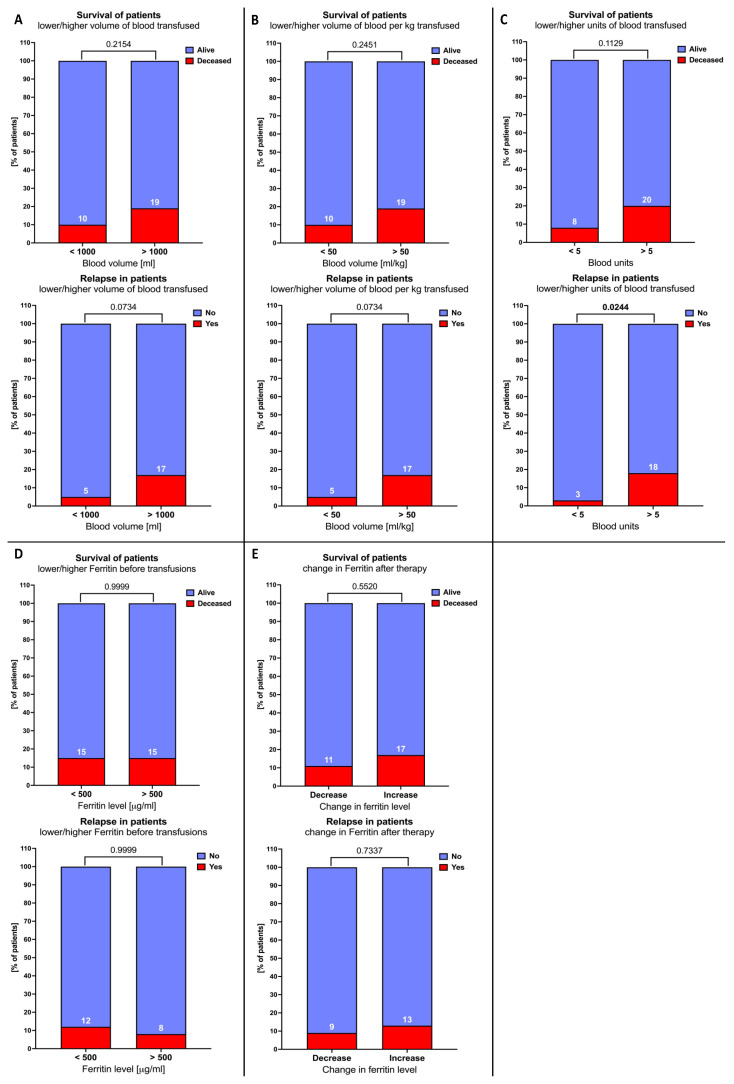
Distribution of relapse and death incidents in lymphoma and solid tumor patients. Chi-square analysis was performed individually, focusing on transfusion-related parameters: total blood volume transfused (**A**), blood volume used per kilogram (**B**), total blood units transfused (**C**), pre-treatment ferritin levels (**D**), and direction of ferritin change in therapy (After versus T0 time point) (**E**). Data show the frequency of death/relapse incidence in specific subgroups, and exact *p* values are provided.

The studied patients were further stratified on the basis of transfusion-related parameters. In accordance, no significant differences were reported in the context of death incidence among the compared subgroups. However, substantial variations were found considering the frequency of relapsed patient incidence. We found, inter alia, that subjects with transfused blood exceeding 5 units showed a higher frequency of relapse (18% versus 3%; *p* = 0.0244) (Figure 5C). A similar tendency was reported in reference to total blood volume or per kilogram transfused. In accordance, groups with transferred blood volumes exceeding 1000 mL or 50 mL/kg comprised a higher percentage of relapsed patients (17% versus 5%; *p* = 0.0734) (Figure 5A,B). Noteworthily, the initial level of ferritin or its change in the course of therapy had no influence on the frequency of death or relapse incidence (Figure 5D,E).

### 3.5. Survival of Patients Subjected to Blood Transfusion Procedures in Lymphoma and Solid Tumor Groups

**Figure 6:**
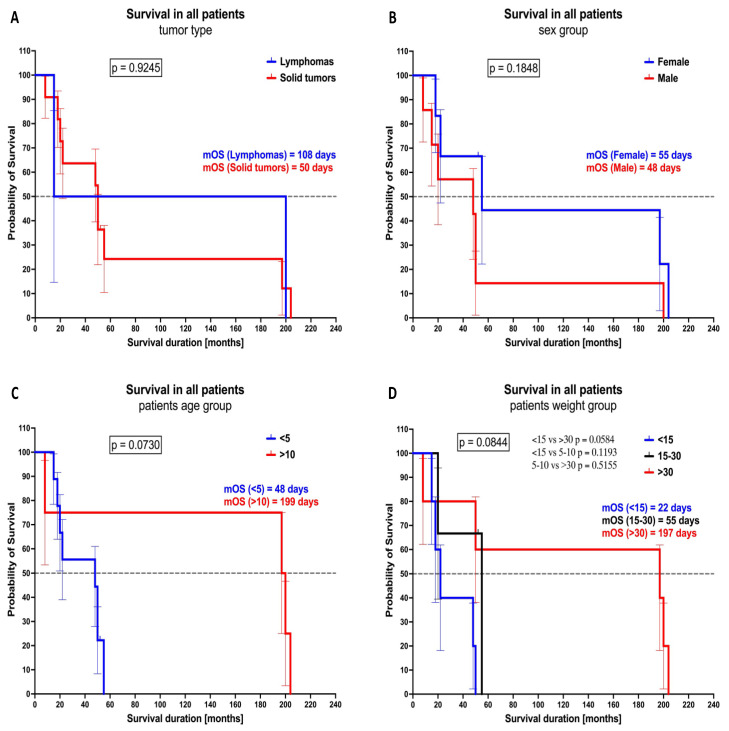
Significance of basic clinical data in reference to studied patients’ survival. Survival curves of the patients analyzed in the context of tumor type (**A**), sex (**B**), age (**C**), and weight (**D**). Data are presented as survival percentages during therapy, with log-rank test results and median overall survival indicated (mOS).

Analysis of survival curves did not reveal substantial differences when tumor type- or sex-based stratification was implemented. Nevertheless, we found that patients older that 10 years old (median overall survival (mOS) = 199 days) had longer survival rates compared to subjects younger than 5 years old (mOS = 48 days) (*p* = 0.0730). In addition, patients weighing above 30 kg (mOS = 197 days) also showed prolonged survival periods versus subjects weighing below 15 kg (OS = 22 days) (*p* = 0.0584) (Figure 6A–D).

**Figure 7:**
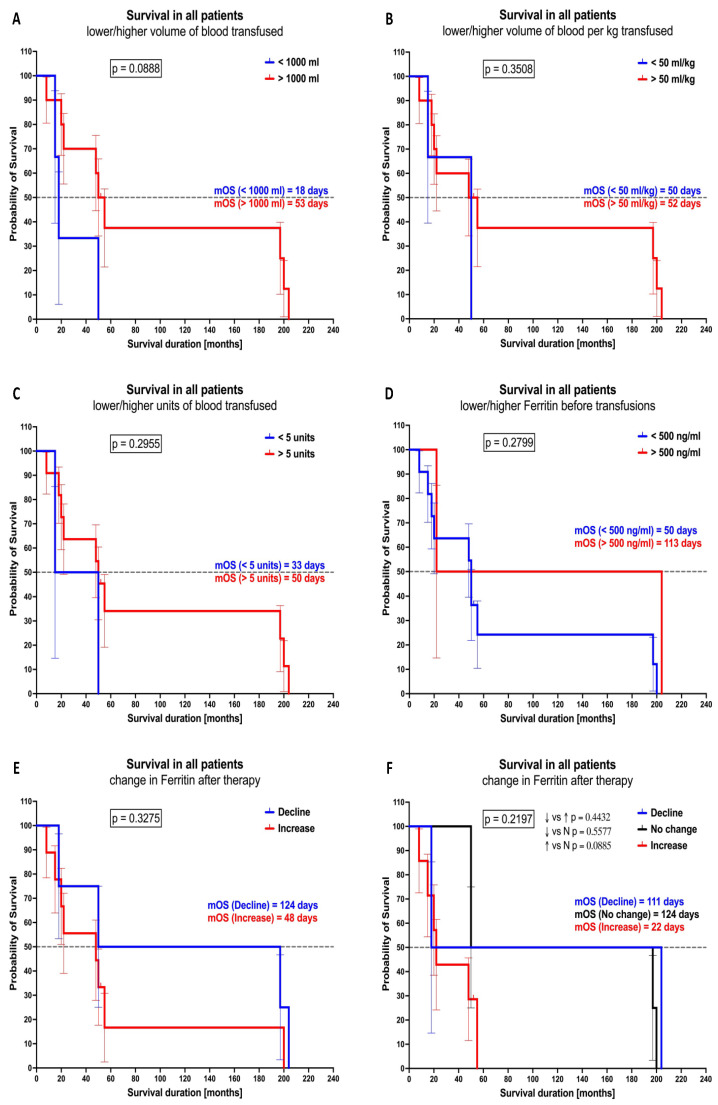
Significance of ferritin- and transfusion-related parameters’ influence on survival of the studied patients. Survival curves of the patients analyzed in the context of total blood volume transfused (**A**), blood volume used per kilogram (**B**), total blood units transfused (**C**), pre-treatment ferritin levels (**D**), and direction of ferritin change in therapy (After versus T0 time point) (**E**,**F**). Data are presented as survival percentages in therapy, with log-rank test results and overall survival indicated (OS).

We did not find statistically significant differences between survival curves when patients were grouped on the basis of transfusion-related parameters and ferritin concentrations. However, subjects with higher blood volumes transfused (>1000 mL) seemed to have better survival rates (mOS = 53 days) compared to the opposite group (mOS = 18 days) (*p* = 0.0888) (Figure 7A). No substantial effects of the basal ferritin levels or their changes over the course of therapy were demonstrated in the context of the patients’ survival. Interestingly, additional stratification of ferritin changes in time revealed that patients with increased levels after therapy had worse survival (mOS = 22 days) versus subjects with no change reported after treatment discontinuation (mOS = 124 days) (*p* = 0.0885) (Figure 7F).

### 3.6. Ferritin Concentration and Transfusion Parameters in High-Risk Group Patients

Ferritin concentrations significantly increased in children diagnosed with higher tumor stages (IV) in the course of treatment when compared to the ones with lower-stage (I–III) tumors (delta ferritin: 39.0 vs. 2.3, *p* = 0.0390). In the group with higher-stage tumors, the final concentrations, as well as delta ferritin, were higher compared to those diagnosed with lower-stage tumors. In all three evaluated transfusion parameters, we found significant differences between the evaluated subgroups (Table 3).

## 4. Discussion

Serum ferritin concentrations undergo changes during antineoplastic treatment due to coexisting clinical situations. Alterations in iron metabolism between cancer and non-malignant tissues are valuable and promising treatment targets [38]. Elevated SF at the moment of diagnosis of solid tumors and lymphomas is mainly associated with the ongoing neoplastic process and accompanying inflammation [2,39,40,41]. Normalization of serum ferritin during treatment is connected with the remission of the oncologic process [2,11,21]. Some childhood solid malignancies are especially connected with elevated ferritin concentrations at diagnosis, like high-stage lymphomas, neuroblastomas, and Hodgkin lymphomas [11,17,18,21,29,42,43,44,45]. High ferritin concentrations were considered a highly predictive marker for remission and a stratifying tool in patients with high-stage NBL [17,42,46]. In the III and IV stages of lymphoblastic lymphomas, considering biochemical markers according to Ann Arbor staging, Hagberg et al. proved that a high ferritin concentration, among other parameters, is associated with worse survival. In Hodgkin lymphoma, high ferritin levels were associated with the presence and intensity of B symptoms, but did not influence the treatment results [43]. 

Age is a strong determinant of serum ferritin. Differences in iron metabolism through the development period result in varied ferritin ranges at a specific age. High SF can be observed in newborns and infants with greater gestational ages and birth weights, as well as those with clinical conditions suggestive of placental dysfunction, infection, and inflammation [47]. In healthy toddlers, SF achieves ranges similar to adults, while in teenagers, we expect lower SF concentrations due to intensive growth and blood loss with the menstrual cycle [27]. Thus, interpretation of SF increases in childhood malignancies can be hampered because of physiological age differences. In our analysis, at the moment of diagnosis, patients older than 10 years old demonstrated significantly higher ferritin concentrations in the blood compared to the subgroup of children below 5 years old and those between 5 and 10 years of age.

Iron load supply in children treated for oncological diseases finished with the intensive treatment, and then, some of the iron stores could be used for the growth processes [27,32,48]. This is the reason for the slow decrease in the elevated SF with the time of observation after the cessation of the treatment, although when strongly elevated, even throughout intensive growth and puberty, they do not normalize. In all analyses performed, we found the ferritin levels to normalize back to the values from before the therapy shortly after its discontinuation. Analysis of a cohort of 214 childhood cancer survivors pointed to greater treatment intensity as the reason for the increased need for transfusions and, thus, the increased risk for iron overload in this group. Ruccione et al. indicated IO, next to chemotherapy toxicities, as a significant late side effect of childhood malignancy which requires organ function monitoring and sensitizing childhood cancer survivors to potential additional liver or pancreatic damage caused by their lifestyle [27,32].

Analysis of the post-transfusion overload on ferritin concentration in Hodgkin lymphoma or solid tumors diagnosed early is difficult due to the very rare indication for blood therapy [14,29]. Patients diagnosed at high stages of solid tumors (III and IV), who have poor prognoses, require more intense treatment and, as a consequence, frequent PRBC transfusions. Post-transfusion iron overload is responsible for the high SF concentrations at the end of the therapy in those patients. Our results corroborate the findings of a retrospective analysis on 92 HR-NBL patients performed by Silverstein et al., who found that high-risk NBL patients, especially those treated with therapy combined with autologous stem cell transplantation, are at high risk of developing iron overload as a result of increased transfusion requests [49]. Moroz et al., similarly to Morgernstern et al., pointed at the important role of ferritin and LDH in high-risk patients of neuroblastoma and encouraged reconsidering utilizing these parameters as a stratification tool [42,46,50]. The selection of patients who will already require chelation therapy due to multiple transfusions at the onset of therapy should result in more restrictive indications for blood transfusions in order to avoid subsequent IO.

Post-transfusion iron overload and SF fluctuations through the course of antineoplastic therapy have been widely discussed in leukemic patients due to multiple transfusions [24,51]. Our previous analysis on 135 children treated for acute lymphoblastic and acute myeloid leukemia showed that patients with higher concentrations of SF at the beginning of treatment exhibit a tendency for higher ferritin concentrations at the end of the treatment, with different reasons for the SF elevation measured at selected time points [52]. Our current analysis proved this phenomenon to be additionally present in patients with solid tumors.

Noteworthily, our findings show that patients with a higher number of transfused blood units and mL/kg of transfused blood represented higher proportion of the relapsed patient group, but were also diagnosed at higher tumor stages, with stage IV neuroblastoma as the most frequent diagnosis. Interestingly, neither the transfusion parameters nor the ferritin level influenced the incidence of deaths among our studied patients. Similarly to Zekavat et al., who determined a cutoff value for predisposition to iron overload of 28.3 mL/kg of transfused blood in leukemic patients, we proved that transfusion-related parameters are an objective marker of IO in lymphomas and solid malignancies [51].

In the course of therapy, a comparable increase in ferritin was observed whether tumor type-, sex-, or weight-based stratification was applied, reaching maximal values at around the 12th to 15th month of treatment. SF changed during treatment as a result of an increasing volume of transfused blood in our patients and has been significantly affected by transfusion-related parameters at the time of therapy. Only subjects treated with more than 50 mL/kg blood volume per kilogram or more than 5 units of blood achieved statistically significant rises in ferritin concentrations. The highest values of SF, which were also higher compared to the opposite subgroup, were found at the 12th and 15th months of treatment.

Patients younger than 5 years old demonstrated the most stable gradual increases in ferritin concentration up to the maximum levels reported at the 15th month of therapy, with no changes reported at all analyzed timepoints in the group older than 10 years of age. According to Ruccine et al., younger children are more prone to developing transfusion-related IO compared to older ones [27,32]. Interestingly, patients with lower basal ferritin levels (below 500 ng/mL) were the only group responding significantly to the therapy, with an increase in ferritin over time. No changes were reported in subjects with pre-treatment levels of over 500 ng/mL. Noteworthy, the ferritin values achieved at the 15th month of therapy and, to a lesser extent, those found after its discontinuation correlated moderately with transfusion-related parameters: total blood volume, blood volume per kilogram, and blood units used. Those associations seemed to be more pronounced within the group of solid tumor patients compared to those diagnosed with lymphomas. In addition, only in lymphoma subjects did show predominantly strong negative correlations of ferritin changes in therapy with the age and weight of the tested patients. Patients exhibiting increases in ferritin throughout the course of therapy seemed to be associated with worse survival compared to patients with no change in ferritin between the discontinuation of treatment and T0 timepoints. Our data are in close relation with a recent study on ferritin and acute leukemia patients after chemotherapy, where high levels of that protein were associated with worse overall and event-free survival. Importantly, the same group demonstrated substantial reversed influence on survival of the soluble hemojuvelin—another iron metabolism marker [53].

We are aware of limitations that may be associated with our work. Firstly, we were not able to obtain or analyze other markers of iron metabolism such as hepcidin, iron, or the even more recently mentioned soluble hemojuvelin [53] at particular points of analysis. However, our estimation of iron concentration during periods between antineoplastic treatment might not be objective, mainly because of the non-stable characteristic of iron. Secondly, coexisting infections due to impaired immunity influence SF. We tried to avoid this discrepancy by omitting ferritin evaluation during inflammation and proof of C-reactive protein or procalcitonin increase. Thirdly, there are more objective methods of assessing post-transfusion IO, for example, T2 magnetic resonance imaging of the liver and heart, but serum ferritin, with its wide availability and low cost, remains a useful and indispensable tool. Moreover, the number of analyzed patients was limited by excluding those who obtained fewer than two blood transfusions, so we excluded a significant group of patients diagnosed with stage I and II malignancies.

## 5. Conclusions

To conclude, our study did not show the influence of high SF at diagnosis on the survival or on the relapse in children with lymphomas and solid tumors. Nevertheless, patients diagnosed with the highest SF represented the group with most of the relapsed patients. Here, we also revealed no substantial differences in blood ferritin levels in the context of tumor type-, sex-, and transfusion-related parameters. As suggested previously, subjects 5 years old and below had lower SF compared to older groups. Those patients had the highest increases in SF as a response to the blood transfusions. In addition, those changes seemed be closely related to the lower SF levels at admission. Importantly, we found that cessation of the therapeutic protocol was associated with fast decline and normalization of this parameter. We further revealed that higher pre- and post-treatment levels of SF were linked to the group of patients at high-risk stages. Despite no significant influence of the SF levels on the survival of the studied tumor patients, that parameter might be useful in monitoring and predicting the efficacy of therapy with blood transfusions. Further studies are required to validate SF’s efficacy for screening when implemented in the clinical setting.

## Figures and Tables

**Table 1:** Characteristics of the analyzed group.

Basic Characteristic of the Patients		
Study groups	Lymphomas	Solid tumors
Tumor types and patient numbers	Hodgkin lymphoma = 10 Non-Hodgkin lymphoma = 12	Soft tissue sarcoma = 14 Bone tumors = 10 Neuroblastoma = 16 Wilms tumor = 8 Germinal tumor = 8 Hepatoblastoma, hepatocarcinoma = 5 Others (brain tumor, histiocytosis) = 6
Age (years)	3.9 years (1.4; 14.1)	
	12.2 years (3.1; 15.6)	3.13 years (1.4; 12.2)
Sex (male%/ female%)	60%/40% (53/35)	
	73%/27% (16/6)	56%/44% (37/29)
Age within sex groups		
Male (3.8 years)	5.4 years (2.3; 15.1)	3.1 years (1.4; 11.6)
Female (4.5 years)	14.7 years (13.6; 15.8)	3.1 years (1.0; 13.2)
Patient distribution in age groups		
<5 years (54.6%)	7/22 (31.8%)	41/66 (62.1%)
5–10 years (10.2%)	3/22 (13.6%)	6/66 (9.1%)
>10 years (35.2%)	12/22 (54.6%)	19/66 (28.8%)

**Table 2:** Transfusion parameters in the analyzed group. Data are presented as medians, and interquartile ranges are indicated (25th and 75th percentile).

	Median [25th and 75th Percentile]
TOTAL (*n* = 88)	
A. Total amount of transfused blood (mL)	1080 [540; 2730]
B. Total amount of transfused blood per kilogram of body weight (mL/kg)	53.85 [26.80; 99.73]
C. Total number of transfusions (units)	5 [3.00; 13.75]
LYMPHOMAS (*n* = 22)	
A. Total amount of transfused blood (mL)	900 [543; 1898]
B. Total amount of transfused blood per kilogram of body weight (mL/kg)	36.60 [13.50; 81.25]
C. Total number of transfusions (units)	3 [2; 8.5]
SOLID (*n* = 66)	
A. Total amount of transfused blood (mL)	1260 [525; 2938]
B. Total amount of transfused blood per kilogram of body weight (mL/kg)	57.65 [31.60; 116.40]
C. Total number of transfusions (units)	5.5 [3.0; 14.0]

**Table 3:** Comparison of ferritin concentration and transfusion parameters between patents diagnosed at stage IV and stages I–III. *p* values indicate differences between risk groups (IV vs. I–III), and asterisks highlight significant differences in ferritin between diagnosis and finishing time point. Data are presented as medians, and interquartile ranges are indicated (25th and 75th percentile).

	STAGE IV	STAGE I–III	*p*
*n*	33	55	
deceased	11/33	2/55	*p* = 0.0014
age of diagnosis	4.98 [1.20; 14.99]	3.77 [1.49; 12.89]	*p* = 0.5364
ferritin at diagnosis	89.7 * [40.9; 218.4]	84.5 [48.0; 221.7]	*p* = 0.9917
ferritin at finish	198.4 * [69.5; 499.5]	99.0 [68.9; 330.3]	*p* = 0.0738
delta ferritin (finish—diagnosis level)	39.0 [−15.7; 305.0]	2.3 [−33.9; 40.3]	*p* = 0.0390
total volume of transfused blood (mL)	2280 [960; 3720]	720 [420; 1500]	*p* < 0.0001
mL/kg of transfused blood	77.4 [50.1; 189.9]	36.0 [22.9; 58.7]	*p* < 0.0001
number of transfusions	12 [4; 19]	4 [3; 6]	*p* < 0.0001

* *p* < 0.05.

## Data Availability

The data presented in this study are available in this article.
